# The Influence of Diet on Ovulation Disorders in Women—A Narrative Review

**DOI:** 10.3390/nu14081556

**Published:** 2022-04-08

**Authors:** Justyna Jurczewska, Dorota Szostak-Węgierek

**Affiliations:** Department of Clinical Dietetics, Faculty of Health Sciences, Medical University of Warsaw, 27 Ciołka Str., 01-445 Warsaw, Poland; jjaniszewska@wum.edu.pl

**Keywords:** female fertility, ovulation disorders, diet, nutrition, insulin sensitivity

## Abstract

Female infertility is commonly due to ovulation disorders. They are mostly related to polycystic ovary syndrome, which is currently viewed as one of the most common endocrine disorders in women of reproductive age. Ovulation-related female fertility is influenced by multiple factors which may include: age, smoking cigarettes, stress, use of psychoactive substances, and physical activity. Moreover, diet-related factors play an important role in the regulation of ovulation. Dietary components that exert a positive influence on ovulation include: carbohydrate products with low glycemic index, plant protein, monounsaturated and polyunsaturated fatty acids, folic acid, vitamin D, antioxidants, and iron. A diet based on the structure of the Mediterranean diet also seems beneficial. Components that have a negative influence mostly include high glycemic index carbohydrates, large amounts of animal protein, saturated fatty acids, and trans fatty acids, which are typically found in the Western model of nutrition. Due to the paucity of studies that presented a direct link between nutrition and the risk of anovulatory infertility, this study aimed to summarize the most recent research on the influence of dietary factors on ovulation disorders and indicate the possibilities of future research.

## 1. Introduction

According to the World Health Organization (WHO), infertility is defined as the inability to conceive despite at least 12 months of regular unprotected intercourse. It is estimated that the problem of infertility may be experienced by even 48 million couples worldwide. The etiology of this disorder may be related to male, female, combined, or idiopathic factors [1]. However, it is believed that female factors are responsible for 35–40% of infertility cases. Female infertility may be due to endocrine disorders, endometriosis, fallopian tube injury, infection, or environmental factors. However, female infertility is commonly associated with ovulation disorders. They are mostly due to polycystic ovary syndrome (PCOS), which is currently viewed as one of the most common endocrine disorders in women of reproductive age [2,3]. It was estimated that 80% of women with PCOS experience ovulation disorders that contribute to problems with conception [4].

The pathomechanism of anovulation in women with PCOS seems to be very complex and may be associated with abnormalities in the secreted gonadotropins and their impaired functioning, which leads to the inhibition of antral follicle development. Common endocrine abnormalities in non-ovulating women with PCOS include elevated serum levels of androgens and luteinizing hormone (LH) with normal or slightly decreased serum follicle stimulating hormone (FSH) levels, which is essential for the proper proliferation of ovarian granulosa cells. Therefore, the role of LH seems to be significant in the pathomechanism of ovulation disorders [5]. It was observed that the follicles of women with PCOS are prematurely sensitive to the action of LH, which results in the suppression of FSH and the impaired development of the dominant follicle. In addition, a high frequency of pulsatile gonadotropin releasing hormone (GnRH) secretion was observed in the group of women with PCOS, resulting in a disturbed LH to FSH ratio. However, it was also observed that antral follicles, which are unable to pass to the pre-ovulatory phase in this group of women, are sensitive to the action of follicle-stimulating hormone. The adjustment of its levels usually restores ovulation and fertility [5,6]. Moreover, the results of a study by Teede et al. [7] additionally indicated the key role of anti-Mullerian hormone (AMH), produced by pre-antral and antral follicles, in the genesis of abnormal GnRH activity and the resulting increase in LH and androgens. Conversely, it is believed that, in addition to neuroendocrine factors, local intraovarian factors also contribute to the arrest of antral follicle development. First of all, elevated blood androgens, affecting numerous metabolic pathways, may lead to the premature arrest of granular cell proliferation, which results in the lack of dominant follicle formation. Another mechanism underlying the causes of ovulation disorders among women with PCOS is related to being overweight and obese [5,6]. Studies by Chavarro et al. [8] and Rich-Edwards et al. [9] revealed a relationship between BMI (Body Mass Index) value and a relative risk of ovulation disorders, as a correlation occurred between excess body weight and LH levels. Moreover, hormone abnormalities in individuals with excessive weight may be exacerbated by associated tissue insulin resistance and hyperinsulinemia, which may potentiate the effect of LH on granular cells in the follicle and increase the chances of premature maturation [5]. Additionally, women with PCOS are characterized by the occurrence of disturbances in adipokines secreted by the adipose tissue, leptin in particular, the concentration of which is significantly elevated in PCOS women with obesity [10]. Leptin levels vary depending on the phase of the menstrual cycle, which means that it plays a significant role in regulating hormonal balance in women and is essential for the course of ovulation [11]. This adipokine stimulates the pituitary gland to secrete LH and may also activate gonadotropin-releasing hormone receptors in the hypothalamus which, in turn, stimulate LH secretion [12]. Therefore, preconception body weight reduction in women with excessive weight is one of the key elements leading to success in conceiving by regulating hormonal balance, decreasing leptin concentration, and inducing spontaneous ovulations [13,14,15]. Furthermore, PCOS women typically present with decreased concentrations of adiponectin, whose action is mainly associated with increasing the sensitivity of tissues to insulin [10]. This adipokine is believed to interact with the hypothalamic–pituitary–gonadal axis and regulate the secretion of GnRH. Adiponectin may also enhance the expression of ovarian insulin receptors and decrease the synthesis of androgens in ovaries, which may lead to an improvement in ovulation, especially in PCOS women [16]. 

Ovulation-related female fertility is influenced by multiple factors that may include: age, smoking cigarettes, stress, the use of psychoactive substances, and physical activity [3,17]. Diet-related factors should also be taken into account, as they play an important role in the regulation of ovulation. Diet and its nutritional components may influence female fertility and ovulation via their effect on metabolic pathways, endocrine profile, and carbohydrate metabolism. Particular significance in the pathogenesis of ovulation disorders is attributed to insulin resistance, which is the most important factor linking anovulatory infertility to nutrition [18]. Another very important factor that increases the risk of ovulation disorders is oxidative stress and chronic inflammation [19]. High levels of inflammatory markers during the menstrual cycle in women are associated with a higher risk of an anovulatory cycle, mainly by increasing oxidative stress in the ovary [20].

Dietary components that have a positive influence on ovulation include: carbohydrate products with low glycemic index, plant protein, monounsaturated and polyunsaturated fatty acids, folic acid, vitamin D, antioxidants, and iron. A diet based on the structure of the Mediterranean diet also seems beneficial due to its anti-inflammatory properties. Components exerting a negative influence mostly include high glycemic index carbohydrates, large amount of animal protein, saturated fatty acids, and trans fatty acids, which are typical of the Western model of nutrition, which is closely related to increased oxidative stress.

The role of the diet and other modifiable factors associated with lifestyle in the prophylaxis of fertility disorders is still under research. However, few studies presented a direct link between nutrition and the risk of anovulatory infertility. Therefore, the aim of the present literature review was to summarize available knowledge about diet and its relation to anovulatory infertility and indicate the possibilities of future research.

## 2. Dietary Patterns

A prospective cohort Nurses’ Health Study II (NHS II), which was conducted in 17,544 women trying to conceive, revealed that diet and nutrients included in it markedly influenced fertility and the reduction of the risk of ovulation disorders. Women from the highest quintile of adherence to diet were at a 66% lower risk of anovulatory infertility compared to women from the lowest quintile. Basing on the results, the so called “fertility diet” pattern was developed. It was characterized by the lower consumption of trans fatty acids with the simultaneous increased supply of monounsaturated fatty acids, higher plant protein content and the presence of high-fat milk products, carbohydrate products with low glycemic index, and high iron content. Moreover, adhering to this dietary pattern was associated with a lower risk of fertility disorders caused by other factors [8]. It was suggested that particular benefits related to consuming the “fertility diet” were observed in women with PCOS, as adherence to the diet was linked to the occurrence of spontaneous ovulation with the overall fertility improvement in this group [21].

The beneficial effect of the Mediterranean diet (MD) on female fertility is also an interesting issue [22,23]. Traditional Mediterranean diet is characterized by the high supply of vegetables, fruit, olive oil, fatty saltwater fish, and whole-grain cereal products. Alcohol consumption should be moderate and red meat consumption should be low [18]. MD has a great potential to reduce inflammation and concentrations of oxidative stress markers, which was linked to the risk of ovulation disorders, particularly in women with PCOS [19]. The results of a study by Fatima et al. [24] showed that patients with PCOS were characterized by low levels of glutathione, vitamins C and E, and significantly increased activity of antioxidant enzymes, such as glutathione peroxidase, glutathione reductase, and glutathione transferase compared to women without PCOS. Chronic, low-grade inflammation in women with PCOS may affect the functioning of the ovaries, interfering with the synthesis and release of sex hormones, follicular maturation, and ovulation [25].

Due to the lack of research to link this diet to the risk of anovulatory infertility, its direct influence on ovulation may not be explicitly confirmed. However, due to its beneficial effect on female fertility and the similarity to the assumptions of the “fertility diet” it may be concluded that it also has a positive influence on ovulation. Additionally, its potentially beneficial effect on ovulation is considerably enhanced by the anti-inflammatory character of the diet [26]. Linoleic acid is considered as a particularly important element of MD. It is a precursor of prostaglandins, which play a significant role in the course of ovulation and increase the response of ovaries to gonadotropin, thereby exerting a positive effect on ovulation [23,27].

The Western dietary pattern is entirely different. It is rich in simple carbohydrates, whose main sources include sugar, sweets, and sweetened beverages, and red and processed red meat. Moreover, it is characterized by the low consumption of fresh fruit and vegetables, whole grain cereals, poultry, and fish. It is a high glycemic index diet, which is additionally rich in saturated fatty acids and trans fatty acids, which increase the risk of anovulatory infertility. The Western dietary pattern is inversely correlated with female fertility through its negative influence on endocrine metabolism and ovarian reserve [17]. According to Hajishafiee et al. [28], lower consistency between nutrition and the Western diet was associated with a 35% lower probability of infertility in women with PCOS. However, due to the lack of research to assess correlations between the Western diet and anovulatory infertility, it may not be explicitly confirmed that the diet increased the risk of ovulation disorders. Nevertheless, considering the correlations between diet and reduced fertility, increased insulin resistance, and a high percentage of components increasing the risk of infertility due to ovulation disorders and aggravating inflammation, it may be assumed that the diet exerts a negative effect on ovulation in women.

## 3. Carbohydrates and Low Glycemic Index Diet

Considering a fertility-promoting nutrition model, one should remember low glycemic index (GI) carbohydrates, as insulin sensitivity and glucose homeostasis are regarded to be some of the most important factors determining female fertility [23]. The mechanism through which the high glycemic index of the diet and high carbohydrate content of the diet contribute to fertility and ovulation disorders results from their influence on tissue sensitivity to insulin. Furthermore, insulin directly influences ovarian function and ovulation via its participation in the response of ovarian follicles to gonadotropin. Therefore, high insulin levels were found to be associated with abnormal ovarian steroid genesis and impaired oocyte development. Moreover, hyperinsulinemia is strongly correlated with hyperandrogenism, which also contributes to the occurrence of ovulation disorders and exacerbates endocrine disorders in women [17,29,30]. Another mechanism linking ovulation disorders to a diet with a high glycemic index and high carbohydrate content appears to be low-grade inflammation. A diet with a high glycemic index and low in dietary fiber was shown to be strongly correlated with inflammation. In particular, fructose is attributed to a strong pro-inflammatory effect. In addition, postprandial hyperglycemia caused by the supply of large amounts of high GI carbohydrates is associated with the intensification of inflammation and oxidative stress through the production of reactive oxygen species [31].

It is believed that both the quality and quantity of carbohydrates have a specific influence on the risk of developing anovulatory infertility. A cohort NHS II study [32] revealed that women in the highest quintile of the total carbohydrate consumption were at a 78% higher risk of anovulatory infertility compared to women from the lowest quintile of the consumption of this macronutrient. The results of these study were confirmed by a systematic review of seven interventional studies, which revealed that the use of a low-carbohydrate diet was associated with a higher ovulation rate [14]. Palomba et al. [33] noted that the combination of a hypocaloric diet providing 45% of energy from carbohydrates with physical activity and clomiphene citrate resulted in a considerable improvement in ovulation rates compared to pharmacotherapy and traditional diet. The results may suggest that reduced carbohydrate supply may be effective in the induction of ovulation in women via the influence on insulin sensitivity. When considering low-carbohydrate diets, it is also worth mentioning the ketogenic diet, which provides less than 20 g of carbohydrates a day, and its impact on the course of polycystic ovary syndrome. The use of this diet is primarily associated with the reduction of body weight, improvement in carbohydrate metabolism, and a significant decrease in insulin resistance and in circulating markers of inflammation [34]. A study conducted by Mavropoulos et al. [35] showed that women with PCOS who followed the ketogenic diet for 24 weeks showed an improvement in the LH/FSH ratio and a reduction of fasting insulin and percentage of free testosterone, which all have a considerable impact on ovulation. However, due to the paucity of research in this area, this diet should be recommended with caution, especially in the case of women of reproductive age.

Apart from the quantity, the quality of the carbohydrates also seems to be a very important factor. A study by Chavarro et al. [32] demonstrated that the consumption of products with low glycemic index (e.g., brown rice, whole grain pasta, and bread) was inversely correlated with the risk of anovulatory infertility, while products with high glycemic index (e.g., white rice, boiled potatoes, or breakfast cereals) had a negative effect on the course of ovulation. 

Special attention is paid to dietary fiber, the source of which are products with low glycemic index. Chavarro et al. [32] demonstrated that fiber consumption increased by 10 g/day was associated with a 44% lower risk of developing ovulation disorders in women aged over 32. Such a correlation was not observed in women aged below 32 years. However, it was found in the BioCycle cohort [36] that the consumption of dietary fiber over the recommended dose was associated with an increased risk of the lack of ovulation. The authors suggested that the observed phenomenon resulted from reduced hormone concentrations due to the high consumption of fiber, especially the water-soluble fraction.

Particular benefits of diets with low glycemic index are observed in women with PCOS. It was noted in this group of patients that the low quality of carbohydrate products and high glycemic index and load were particularly linked to ovulation disorders [37]. It is believed that insulin resistance and hyperinsulinemia are factors that lead to ovulation disorders, endocrine disorders, and abnormal endometrial structure, and, therefore, to infertility in PCOS patients. Hyperinsulinemia may also have a direct negative effect on the development of ovarian follicle in this group of women. It may even inhibit its development, leading to anovulatory cycles [4,38]. Furthermore, a meta-analysis and systematic review of 10 randomized studies revealed that the use of low glycemic index diets was associated with decreased testosterone levels in women with PCOS. It suggests that the beneficial effect of a low GI diet in this group of women was not only associated with the direct influence of carbohydrates on fertility and ovulation, but also with the influence of the diet on hormonal regulation, whose homeostasis determined the normal course of ovulation [39]. According to a randomized study by Sordia-Hernández et al. [40], which included 37 women with PCOS, ovulatory cycles occurred in 24.6% of women consuming a low glycemic index diet. Only 7.4% of women consuming a traditional diet who did not focus on glycemic index had ovulatory cycles. The observed differences in the frequency of ovulation cycles in both diets may result from reduced androgen concentrations and increased tissue sensitivity to insulin being the consequence of consuming a low glycemic index diet.

Another important factor associated with carbohydrate products is the influence of dietary Advanced Glycation End Products (AGE) on female fertility and ovulation. AGEs are formed as a result of the reaction of the amino groups of protein, lipid, amino acid, and nucleic acid with the aldehyde group of reducing carbohydrate, during frying and preparing products rich in carbohydrates and proteins at high temperatures. They are especially characteristic for the Western diet, which is rich in highly processed products, simple sugars, animal protein, and fat. It is believed that they play an essential role in the deregulation of ovarian function and ovulation, because they may accumulate in the granulosa cell layer. Diets high in AGE compounds may disrupt ovarian function, folliculogenesis, and steroidogenesis in particular, contributing to oxidative stress and disrupting hormonal balance. AGEs mainly interfere with LH and FSH action and they lead to ovulation disorders in women with PCOS [41,42].

To sum up, due to the fact that the insulin sensitivity of tissues is one of the more important determinants of the normal course of ovulation, low glycemic index diet plays a significant role in its regulation. Moreover, it seems that limiting carbohydrate supply is also of key importance in the prevention of ovulatory infertility. Therefore, the diet of a woman trying to conceive who has problems related to ovulation disorders should be balanced with regards to both the quantity and quality of carbohydrates provided.

## 4. Plant and Animal Protein

Wholesome protein also constitutes a very important component in the “fertility diet”. However, some studies showed that protein might have a negative effect on fertility, which is mainly related to its source [43,44]. Chavarro et al. [44] demonstrated that women from the highest quintile of total protein consumption were at a 41% higher risk of anovulatory infertility compared to women from lower quintiles of the consumption of this macronutrient. Furthermore, the addition of one portion of meat daily resulted in a 32% increase in the risk of ovulatory disorders. It was shown that protein obtained from red meat and poultry considerably increased the risk of anovulatory infertility, while no negative influence on ovulation was observed in the case of egg and fish protein. A study by Zhang et al. [45], which included 2217 women with PCOS without ovulation and with normal ovulation, revealed that women with ovulation disorders were characterized by a significantly higher share of meat in the diet compared to women with normal ovulation. Moreover, red processed meat was found to have a particularly negative effect on fertility, as its consumption was related to numerous adverse health outcomes. Therefore, its negative effect on ovulation may also be speculated [46]. However, it is worth noting that women characterized by a higher consumption of animal protein also consumed more saturated fatty acids compared to those who consumed smaller quantities of animal protein. They were also less physically active. Therefore, the potential influence of both those factors needs to be considered, as they may intensify the correlation between animal protein consumption and ovulation disorders [44].

An entirely different effect on ovulation was observed in relation to plant protein. The consumption of 5% of energy from plant protein instead of animal protein diminished the risk of anovulatory infertility by over 50%. Furthermore, changing carbohydrates into plant protein also appeared to have a positive effect on ovulation. The consumption of plant protein at the level of 5% of energy requirement instead of carbohydrates was associated with the reduction of the risk of ovulation disorders by as much as 43% [44]. A potentially beneficial effect of plant protein on fertility may be linked to improved insulin sensitivity and lower postprandial secretion of this hormone compared to animal protein [17]. Both types of protein have an entirely different effect on the concentrations of circulating IGF-1 (Insulin-like Growth Factor 1). It was observed that women consuming higher amounts of animal protein had higher IGF-1 concentrations, which was correlated with the occurrence of ovulation disorders and abnormal development of ovarian follicles [18,47]. 

The effect of milk products on ovulation disorders is another interesting issue, as they constitute a significant source of protein in the diet. Milk products are believed to have a toxic influence on fertility due to the high galactose content, which disturbs ovulation in mice and leads to premature ovarian insufficiency [48]. Additionally, their consumption adversely affects hormonal regulation in women [36]. A cohort BioCycle study [49] showed that a higher frequency of anovulation was noted in women consuming higher amounts of cream and yoghurt. However, some authors confirmed a positive influence of milk and milk products on female fertility, regardless of fat content [50,51]. Interesting results were obtained in a cohort NHS II study [52], which showed no correlation between the total consumption of milk products and anovulatory infertility. However, significant differences were observed as regards their influence on ovulation depending on fat content. Increasing the consumption of low-fat milk products by one portion daily was linked to an 11% increase in the risk of anovulatory infertility, while adding one portion of whole milk without increasing energy consumption decreased the risk of ovulatory infertility by over 50%. The authors suggested that differences in the influence of milk products with various fat content on ovulation resulted from the fact that milk products characterized by higher fat content had higher estrogen content and caused lower IGF-1 increase compared to their lean equivalents. Furthermore, a beneficial effect of high-fat milk products on ovulation may be associated with the presence of trans palmitic acid, which seems to increase insulin sensitivity [17]. Moreover, the relationship between the consumption of low-fat and high-fat milk products and anovulatory infertility appeared to be particularly intense in women without typical PCOS clinical manifestations compared to women with such manifestations [52]. 

To sum up, basing on the research, it may be speculated that a higher share of plant protein than animal protein is more beneficial in the context of anovulatory infertility. Due to the lack of research to link the consumption of red meat to anovulatory infertility, it may not be confirmed whether the product increases the risk of ovulation disorders. However, considering the fact that red meat consumption, particularly processed, increases insulin resistance, its negative effect on ovulation may be speculated. Moreover, due to the lack of explicit research results, the influence of milk products on ovulation may not be explicitly confirmed, particularly as regards high-fat products, which are the source of saturated fatty acids, thereby intensifying ovulation disorders.

## 5. Unsaturated and Saturated Fatty Acids

Suitable quality and quantity of consumed fatty acids is of utmost importance in the prophylaxis of fertility disorders. Both insufficient and excessive amount of fat in the diet seem to have a negative effect on fertility. Insufficient fat content in the diet may contribute to the occurrence of abnormal menstrual cycles (prolonged follicular phase, secondary amenorrhea, and longer cycles) [53]. A study by Chavarro et al. [53] revealed that total fat consumption was inversely proportional to the risk of anovulatory infertility. However, after comprising potential confounding factors, the correlations were significantly weaker and statistically insignificant. Conversely, the results obtained by Mumford et al. [54] indicated that high-fat diet triggered increased testosterone synthesis in women, which also affects ovulation. Additionally, it is assumed that high-fat diet disrupts the functioning of the hypothalamic–pituitary–ovarian axis, leading to endocrine disorders and prolonged menstrual cycles, which may also contribute to the development of ovulation disorders in women. This correlation is mostly due to insulin resistance and excessive ovarian and hypothalamic stimulation by insulin [55].

However, the quality, and not the quantity, of fat in the diet seems more important as regards ovulation disorders. It is believed that PUFA (polyunsaturated fatty acid) supplementation exerts a beneficial effect on female fertility through the influence on LH and FSH concentrations, maturation of the dominant follicle, the quality of oocytes, and ovulation induction [56]. Moreover, omega-3 fatty acids regulate the maturation and development of oocytes mostly via the regulation of the PPAR (peroxisome proliferator-activated receptor) receptor. The expression of all three of its isoforms was identified in the ovarian tissue. The expression of PPARγ increases with the growth of the follicle and is subsequently rapidly decreased in response to LH release and ovulation. Furthermore, omega-3 acids stimulate ovulation via the expression of genes and COX-2 (cyclooxygenase 2) activity [57]. It was demonstrated that omega-3 acid supply was associated with higher progesterone concentrations and a lower risk of ovulation disorders. As regards women with PCOS, PUFA acids have a positive effect on metabolic and endocrine parameters. However, their direct influence on ovulation was not observed in this group of women [54]. Mumford et al. [58] demonstrated that the consumption of docosapentaenoic acid, which is structurally similar to eicosapentaenoic acid, was linked to a reduced risk of anovulation in a cohort of healthy and regularly menstruating women. Similar inverse correlations were observed for their polyunsaturated omega-3 fatty acids. However, they were devoid of statistical significance. Furthermore, the consumption of total fat and polyunsaturated fatty acids was unrelated to higher testosterone levels, but it was associated with increased progesterone levels, which promoted the reduction of the risk of anovulation. Monounsaturated fatty acids (MUFA) are also beneficial in the context of fertility mainly by reducing inflammation [58]. Interestingly, Chavarro et al. [53] reported that the consumption of MUFA, total polyunsaturated fatty acids, n-3 PUFA, and n-6 PUFA was not associated with anovulatory infertility.

SFA (saturated fatty acids) and TFA (trans fatty acids) were found to exert a particularly negative effect on ovulation [59]. Interestingly, cohort studies by Mumford et al. [54] and Chavarro et al. [53] revealed no correlation between SFA consumption and the relative risk of anovulation. However, Chavarro et al. [53] observed a correlation between the consumption of TFA contained in sweets, hard margarines and fast food, and ovulation disorders. The replacement of unsaturated fatty acids with saturated fatty acids also has a negative impact on ovulation disorders. The change of 2% of energy obtained from polyunsaturated fatty acids or monounsaturated fatty acids into TFA was associated with a doubled risk of anovulatory infertility. Moreover, each increase of energy obtained from TFA by 2% instead of carbohydrate-derived energy was also associated with anovulatory infertility, and each increase of energy from TFA by 2% was associated with a 73% increase in the risk of ovulation disorders in women. Ghaffarzad et al. [60] conducted a study in women with PCOS and demonstrated that higher concentrations of trans fatty acids in erythrocytes were associated with an increased risk of ovulation disorders in this group of women. A potential correlation between TFA and anovulatory infertility was related to the increased insulin resistance of tissues, inflammatory marker concentrations, and a 40% reduction in the expression of the PPARγ receptor, which were attributed a significant share in the regulation of ovulation.

To sum up, a diet rich in monounsaturated and polyunsaturated fatty acids seems to have a positive effect on ovulation. However, good quality research is necessary in this matter for the explicit identification of a correlation between unsaturated fatty acids and ovulatory fertility. Moreover, the influence of the amount of fat in the diet is dubious, so research is necessary as well. Trans fatty acids also have a negative influence on ovulation. Therefore, diet poor in processed products, sweets, and fast food seems to have a positive effect on the risk of ovulation disorders.

## 6. Alcohol and Caffeine

Caffeine and alcohol seem to have a negative effect on female fertility, as they may particularly increase the risk of ovulation disorders [61]. Chavarro et al. [61] demonstrated that the consumption of energy drinks containing caffeine was related to a 47% increase in the risk of anovulatory infertility in women consuming at least two or more caffeine-containing beverages compared to women who drank less than one beverage with caffeine per week. Various hypotheses have been developed as regards the possible mechanisms of the potential influence of caffeine on reproduction, but the mechanisms have not been elucidated yet. Caffeine consumption may affect ovulatory fertility via the influence on reproductive hormone concentrations (e.g., decreased levels of estradiol), changes in hormone metabolism, and the activity of ovaries. Due to its interaction with sex hormones, caffeine consumption may negatively affect the length of the cycle. High caffeine intake (>300 mg/day) may even inhibit ovulation, but the mechanism is still unclear [62]. 

Despite a potentially negative influence of caffeine on fertility, some research showed no correlation between drinking tea and coffee and female fertility [63,64]. Chavarro et al. [60] revealed no correlation between the consumption of caffeine from tea and coffee and the risk of anovulatory infertility. A potentially positive influence of caffeine on ovulation may result from its effect on tissue insulin sensitivity and carbohydrate metabolism, which modulates the process of ovulation to a considerable extent [65]. 

The effect of alcohol on ovulation is currently under research with the results being contradictory. Differences in the results may mostly be due to the type of consumed alcohol, health status, and other confounding factors [66]. The negative influence of alcohol consumption on female fertility was mainly due to its effect on hormonal regulation, menstrual cycle, ovarian reserve, oocyte maturation, and ovulation [17,67,68]. Alcohol consumption, leading to increased estrogen concentrations and decreased FSH, inhibits folliculogenesis and ovulation [69]. According to Chavarro et al. [61], women consuming about 10 g or more alcohol daily (about >1 drink a day) were at an almost 50% higher risk of anovulatory infertility compared to women who denied drinking alcohol. The correlation was particularly intense in the case of spirit drinks.

To sum up, due to the lack of unambiguous results, it may not be stated that caffeine consumption disturbs ovulation in women. Seemingly, tea and coffee consumption does not affect ovulation, while energy drinks containing caffeine may considerably disturb ovulation in women. Moreover, alcohol consumption has a particularly unfavorable effect in the context of ovulatory infertility. Due to the negative effect of alcohol on other fertility parameters, it should be eliminated from the diet of women trying to conceive.

## 7. Vitamins and Minerals

Appropriate supply of vitamins and minerals also has a positive influence on female ovulatory fertility. A significant role is attributed to group B vitamins (B6, B12, and folic acid in particular), antioxidant vitamins (A, C, E), vitamin D, and iron. A study by Chavarro et al. [70] demonstrated that the consumption of multivitamin supplements at least three times a week was associated with the reduced risk of anovulatory infertility. The correlation seems to be mostly related to folic acid. It was confirmed that the consumption of 700 μg of folic acid daily reduced the risk of ovulation disorders by 40–50%. Another cohort BioCycle Study by Gaskin et al. [71] including 259 healthy women aged 18–44 showed that folic acid supplementation was inversely correlated with the risk of anovulation. Women from the highest tertile of folate consumption (270.6 μg/d) had 64% lower chances of developing anovulation compared to women from the lowest tertile (100.9 μg/d). A similar correlation was observed as regards the consumption of cereal products fortified with folic acid with the observed correlation between tertiles being non-linear. Interestingly, such a correlation was not observed with reference to the consumption of folates with food. It might have been due to the fact that synthetic folic acid is more easily absorbed in the digestive tract compared to its natural equivalents.

The mechanism through which folic acid exerts a beneficial effect on female fertility is mostly related to its influence on oxidative stress and the production of proinflammatory cytokines, which may have a significant effect on ovulation and the development of oocytes. Another possible mechanism through which folic acid influences the course of ovulation is related to the lower response of ovaries to FSH stimulation in the case of low folate concentrations in the blood serum [71]. However, its influence on homocysteine concentrations seems to be crucial. It regulates homocysteine concentrations with vitamin B6 and B12, so their supply seems to be of key importance for female fertility [17]. A cohort study including 259 regularly menstruating women who used no hormonal contraceptives or diet supplements revealed a correlation between higher homocysteine concentrations and a 33% increase in the risk of anovulation. Moreover, a higher ratio of folic acid to homocysteine reduced the risk of anovulation by 10%. The correlation was mostly due to the fact that homocysteine influenced the concentrations of reproductive hormones during individual phases of the cycle, which was important in the context of ovulation [72]. 

Antioxidants also seem very important for ovulation, as oxidative stress was found to increase the risk of anovulatory infertility [17]. Possible mechanisms of action of antioxidants on female fertility include improved blood flow in the endometrium, reduced reproductive hormone concentrations, increased tissue sensitivity to insulin and the influence on ovulation, prostaglandin synthesis, and steroid genesis [73]. Furthermore, vitamin C found at high concentrations in oocyte cytosol participates in collagen synthesis, which is important for the growth of the Graafian follicles, ovulation, and the luteal phase [17]. Considering the influence of vitamins on female fertility, a particular role is attributed to vitamin D. Vitamin D may take part in the modulation of female reproductive functions, as its receptors are present in numerous tissues of reproductive organs, such as the ovaries, endometrium, and placenta. Moreover, vitamin D influences a number of endocrine processes and the steroid genesis of reproductive hormones. Furthermore, it may induce oocyte maturation and ovulation. It also influences carbohydrate metabolism and insulin sensitivity of tissues, which may contribute to the modulation of ovulation [17]. A randomized placebo-controlled clinical trial included 186 women in whom ovulation was induced with clomiphene citrate and combined with vitamin D supplementation. Vitamin D supplementation significantly improved ovulation rates (92.5% of women in the treatment group vs. 78.5% in the control group had successful ovulation) [74]. Similar results were obtained in a different randomized study including women in whom ovulation was induced with clomiphene citrate and who were also administered vitamin D or coenzyme Q10. The study revealed that both supplements significantly increased the indices of ovulation in PCOS women resistant to clomiphene citrate treatment. Furthermore, a marked improved endocrine profile was noted in both groups [75].

As regards minerals, iron seems the most important. Its deficiencies are frequently observed in women of reproductive age as a consequence of menstruation-related loss. The use of non-heme iron and iron supplements was inversely correlated with the risk of ovulation disorders in women in the cohort NHS II study. The correlation was probably due to the presence of transferrin in the ovaries. It influences the development of ovarian follicles and female gametes [76]. Another cohort BioCycle Study [77] was carried out to assess the correlation between the consumption of minerals and the risk of ovulation disorders. The correlation was only confirmed between low sodium, selenium, and manganese consumption. The consumption of sodium < 1500 mg, selenium < 55 μg, and manganese < 1.8 mg was associated with an increased risk of anovulation compared to suitably higher consumption. No influence on ovulation was observed as regards other minerals.

**Figure 1:**
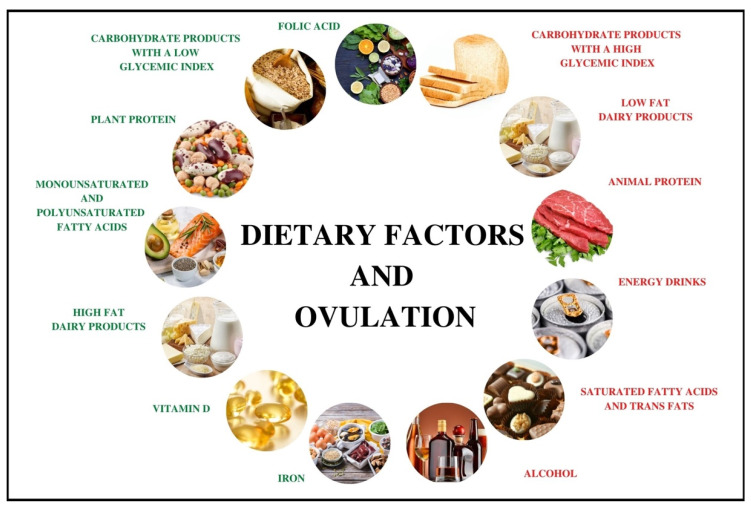
The influence of dietary factors on the risk of ovulation disorders. Factors with a positive influence on ovulation are presented in green, while those which increase the risk of ovulation disorders are presented in red. Ovulation is positively influenced by components typical of the Mediterranean diet (e.g., low glycemic index carbohydrates, plant protein, and unsaturated fatty acids), while components typical of the Western diet have a negative effect (e.g., animal protein, carbohydrates with a high glycemic index, and saturated fatty acids).

To sum up, a diet rich in vitamins and minerals exerts an extremely significant effect on ovulation. Particular significance is attributed to group B vitamins, which participate in the regulation of homocysteine concentrations. Moreover, the role of antioxidant vitamins and vitamin D seems very important. The effect of iron on the regulation of ovulation also seems promising, but there is a paucity of studies to link the consumption of iron to ovulation. The summary of the influence of particular dietary factors on the risk of ovulation disorders was presented in the Table 1 and Figure 1.

## 8. Conclusions

Adherence to a balanced diet with all necessary nutrients has a considerable influence on female fertility and the risk of ovulation disorders. An adequate supply of plant protein, unsaturated fats, and low glycemic index carbohydrates is of high importance. Moreover, it is crucial to provide the appropriate amount of vitamins and minerals, mostly including folic acid, vitamin D, antioxidant vitamins, and iron. Additionally, the supply of saturated fatty acids, trans fatty acids, and high glycemic index carbohydrates should be limited. However, due to the lack of randomized clinical trials in this area, especially as regards a group of women with polycystic ovary syndrome, the importance of individual components in ovulation may not be clearly confirmed. It is necessary to perform more high-quality research comprising the significance of individual nutrients, food products, and the whole structure of the diet in order to develop a model of nutrition that will be helpful in the treatment of women with ovulation disorders.

## Figures and Tables

**Table 1:** Dietary patterns and the risk of anovulation.

First Author/Reference Number	Year	Study Design	Sample	Result
Dietary pattern				
Chavarro et al. [8]	2007	A prospective cohort study (NHS II)	17,544 women, aged 25–42 years	Higher adherence to the FD was associated with a lower risk of anovulatory infertility compared to the lowest adherence (RR 0.34 (95% CI 0.23–0.48) vs. RR 0.68 (95% CI 0.52–0.89); *p* < 0.001).
Carbohydrates and low glycemic index diet				
Chavarro et al. [32]	2009	A prospective cohort study (NHS II)	18,555 women, 25–42 years	An increase in cereal fiber intake by 10 g/day was associated with a 44% lower risk of anovulatory infertility among women older than 32 (RR 0.56 (95% CI 0.34–0.93); *p* = 0.02). The risk of anovulatory infertility was 78% higher in women from the highest quintile of total carbohydrate intake (60% of calories) compared to women from the lowest quintile (42% of calories) (RR 1.78 (95% CI 1.14–2.78); *p* = 0.005). A linear trend towards a higher risk of anovulatory infertility with increasing carbohydrate intake (*p* trend = 0.005). The risk of anovulatory infertility was 92% higher in women from the highest quintile of glycemic load compared to women from the lowest quintile (RR 1.92 (95% CI 1.26–2.92); *p* = 0.01). The risk of anovulatory infertility was 55% higher in nulliparous women from the highest quintile of glycemic index compared to women from the lowest quintile (RR 1.55 (95% CI 1.02–2.37); *p* = 0.05). Dietary glycemic index was positively associated with the risk of anovulatory infertility among nulliparous women (*p* interaction = 0.02).
Gaskin et al. [36]	2009	A prospective cohort study (The BioCycle Study)	250 women, aged 18–44 years	Each 5 g/day increase in total fiber intake was associated with a 78% increased risk of an anovulatory cycle (RR 1.78 (95% CI 1.11–2.84); *p* < 0.05). Soluble fiber had a stronger, positive association with an increased risk of anovulation (OR 6.73 (95% CI 1.18–38.26) than insoluble fiber (OR 2.15 (95% CI 1.22–3.77) and fruit fiber (OR 3.05 (95% CI 1.07–8.71); *p* < 0.05). Dietary fiber consumption was positively associated with incidents of anovulation (*p* = 0.004), with an OR of 10.98 (95% CI: 1.5, 80.5) for women at or above the DRI (≥22 g/day) compared to the lowest DRI (≤10 g/day).
McGrice et al. [14]	2017	A Systematic Review of 7 intervention studies	Infertile women with obesity, aged > 18 years	The use of low-carbohydrate diet (less than 45% of total energy obtained from carbohydrates) was associated with a higher ovulation rate (*p* < 0.05) compared to the usual diet.
Palomba et al. [33]	2010	A randomized controlled trial	96 PCOS women with obesity, aged 18–35 years with anovulatory infertility and known CC resistance	The intervention (structured exercise + 35% protein, 45% carbohydrate, 20% fat diet with calorie deficit + CC) was effective in increasing probability of ovulation under CC treatment.
Sordia-Hernández et al. [40]	2015	A randomized controlled clinical trial	40 patients with the diagnosis of PCOS, infertility, and anovulation, mean age 26 years	24.6% (14/57) of the cycles were ovulatory in women who consumed a low glycemic index diet. In those who consumed a normal glycemic index diet, only 7.4% (4/54) of the cycles were ovulatory (*p* = 0.014).
Plant and animal protein products				
Chavarro et al. [52]	2007	A prospective cohort study (NHS II)	18,555 women, 25–42 years	The risk of anovulatory infertility was 27% lower in women from the highest quintile of intake of high-fat dairy products (≥1 servings/day) compared to women from the lowest quintile (≤1 servings/week) (RR 0.73 (95% CI 0.52–1.01); *p* < 0.05). Adding one daily serving of full-fat milk without increasing energy intake was associated with a reduction in the risk of anovulatory infertility by 63% (RR 0.37 (95% CI 0.19–0.70), *p* = 0.002). An inverse association between dairy fat intake and anovulatory infertility (*p* trend = 0.05). The risk of anovulatory infertility higher by 71% in women from the highest quintile of intake of low-fat dairy products (especially yoghurt and sherbet/frozen yoghurt) (one serving a day) compared to women from the lowest quintile (≤1 servings/week) (RR 1.71 (95% CI 1.24–2.77); *p* = 0.002).
Chavarro et al. [44]	2008	A prospective cohort study (NHS II)	18,555 women, 25–42 years	Consuming 5% of energy from plant protein rather than from carbohydrates was associated with a 43% lower risk of anovulatory infertility (RR 0.57 (95% CI 0.32–1.00); *p* = 0.05). Consuming 5% of energy from vegetable protein rather than from animal protein was associated with a 52% lower risk of anovulatory infertility (RR 0.48 (95% CI 0.28–0.81); *p* = 0.007). The risk of anovulatory infertility was 41% higher in women from the highest quintile of total protein intake (23.1% of calories) compared to women from the lowest quintile (15.4% of calories) (RR 1.41 (95% CI 1.04–1.91); *p* = 0.02). The risk of anovulatory infertility was 39% higher in women from the highest quintile of animal protein intake (18.5% of calories) compared to women from the lowest quintile (10.2% of calories) (RR 1.39 (95% Cl 1.01–1.90); *p* = 0.03). Adding one serving of meat per day was associated with a 32% higher risk of anovulatory infertility (RR 1.32 (95% CI 1.08–1.62); *p* = 0.01). Adding one serving of chicken or turkey per day was associated with a 53% greater risk of anovulatory infertility (RR 1.53 (95% CI 1.12–2.09); *p* = 0.01). Consuming 5% of total energy intake as animal protein instead of from carbohydrates was associated with 19% greater risk of anovulatory infertility (RR 1.19 (95% CI 1.03–1.38); *p* = 0.02).
Kim et al. [49]	2017	A prospective cohort study (The BioCycle Study)	259 healthy, regularly menstruating women, aged 18–35 years	Associations between intakes of >0 servings of yoghurt (RR 2.1 (95% CI 1.2–3.7) and cream (RR 1.8 (95% CI 1.0–3.2) and a higher risk of sporadic anovulation compared to no intake.
Zhang et al. [44]	2020	A prospective cohort study	2217 infertile women with PCOS (with ovulation and without), aged > 18 years	PCOS women with anovulation had a higher rate of meat favorable diet than PCOS women with ovulation (54.60% vs. 41.30%, RR 1.69 (95%CI 1.28–2.23), *p* < 0.01).
Unsaturated and saturated fatty acids				
Chavarro et al. [53]	2007	A prospective cohort study (NHS II)	18,555 women, 25–42 years	Each 2% increase in the intake of energy from *trans* unsaturated fats, rather than from carbohydrates was associated with a 73% higher risk of anovulatory infertility (RR 1.73 (95% CI 1.09–2.73); *p* = 0.02). Obtaining 2% of energy intake from trans fats rather than from n-6 polyunsaturated fats was associated with a 79% higher risk of anovulatory infertility (RR 1.79 (95% CI 1.11–2.89); *p* = 0.02). Obtaining 2% of energy from trans fats rather than from monounsaturated fats was associated with a more than doubled risk of anovulatory infertility (RR 2.31; (95% CI 1.09–4.87), *p* < 0.05).
Ghaffarzad et al. [60]	2014	A case-control study	29 women with PCOS, aged 19–35 years	Higher concentrations of trans fatty acids (trans linoleate) in erythrocytes were associated with an increased incidence of ovulation disorders in this group of women (OR 1.218 (95% CI 1.016–1.46); *p* = 0.033).
Mumford et al. [54]	2016	A prospective cohort study (The BioCycle Study)	259 regularly menstruating, healthy women, aged 18–44 years	The intake of PUFA docosapentaenoic acid (22:5 n–3) was associated with a reduced risk of anovulation (highest tertile compared with the lowest tertile: (RR: 0.42 (95% CI 0.18–0.95); *p* < 0.05).
Alcohol and caffeine				
Chavarro et al. [61]	2009	A prospective cohort study (NHS II)	18,555 women, 25–42 years	Women consuming 2 or more caffeinated soft drinks per day were at a 47% greater risk of anovulatory infertility than women who consumed less than 1 caffeinated soft drink per week (RR 1.47 (95% CI 1.09–1.98); *p* = 0.01). Women consuming 10 g or more of alcohol per day (approximately > 1 drink/day) were at a 47% greater risk of anovulatory infertility than women who did not drink any alcohol (RR 1.47 (95% CI 1.02–2.10), *p* = 0.03).
Vitamins and minerals				
Chavarro et al. [76]	2006	A prospective cohort study (NHS II)	17,544 women, aged 25–42 years	Women who consumed iron supplements were at a significantly lower risk of anovulatory infertility than women who did not use iron supplements (RR 0.60 (95% CI 0.39–0.92); *p* = 0.003). The risk of anovulatory infertility was 47% lower in women from the highest quintile of iron intake (77 mg/day) compared to women from the lowest quintile (11 mg/day) (RR 0.53 (95% CI 0.35–0.82); *p* = 0.003). The risk of anovulatory infertility was 40% lower in women from the highest quintile of nonheme iron intake (76 mg/d) compared to women from the lowest quintile (9.7 mg/d) (RR 0.60 (95% CI (0.39–0.92); *p* = 0.005).
Chavarro et al. [70]	2008	A prospective cohort study (NHS II)	18,555 women, 25–42 years	The risk of anovulatory infertility was 41% lower in women who used multivitamins ≥ 6 times per week compared to women who did not use multivitamins (RR 0.59 (95% CI 0.46, 0.75); *p* < 0.001). The risk of anovulatory infertility was 39% lower in women from the highest quintile of intake of folic acid (1138 μg/day) compared to women from the lowest quintile (243 μg/day) (RR 0.61 (95% CI 0.37, 1.00); *p* = 0.04).
Gaskin et al. [71]	2012	A prospective cohort study (The BioCycle Study)	259 women, aged 18–44 years	Women in the highest tertile of folate consumption (270.6 g/d) had a 64% lower chance of anovulation compared to women in the lowest tertile of folate consumption (100.9 g/d) (OR 0.36 (95% CI 0.14, 0.92); *p* = 0.03).
Kim et al. [77]	1217	A prospective cohort study (The BioCycle Study)	259 regularly menstruating women, aged 18–44 years	Sodium intake < 1500 mg (RR 2.70 (95 % CI 1.00–7.31) and manganese intake < 1.8 mg (RR 2.00 (95% CI 1.02–3.94) were associated with an increased risk of anovulation, compared to higher intakes, *p* < 0.05.
Yahya et al. [75]	2019	A randomized- controlled, open-label study	45 PCOS women, aged 18–40 years	Both dietary supplements (vitamin D3 or CO-enzyme Q10) in combination with CC, significantly improved ovulation rates in clomiphene citrate-resistant women with PCOS.
Rasheedy et al. [74]	2019	A double blind, randomized clinical trial	186 women undergoing the induction of ovulation with CC, aged 25–35 years	Women with PCOS undergoing the induction of ovulation: vitamin D supplementation significantly improved the ovulation rate. More than 90% (92.5%) of women in the treatment group took CC (50 mg) twice daily and vitamin D3 (10,000 IU), and 78.5% in the control group (placebo) had successful ovulation (*p* = 0.007).

Abbreviations: NHS II, Nurses’ Health Study II; RR, relative risk; Cl, confidence interval; FD, fertility diet; OR, odds ratio; PUFA, Polyunsaturated Fatty Acids; PCOS, polycystic ovary syndrome; DRI, dietary recommended intake; CC, clomiphene citrate, IU, international unit.

## Data Availability

Not applicable.
